# Cytomegalovirus-Associated Pancreatitis in Acquired Immunodeficiency Syndrome

**DOI:** 10.1155/1992/71516

**Published:** 1992

**Authors:** Emilio González-Reimers, Francisco Santolaria-Fernádez, Juan L. Gómez-Sirvent, Rafael Méndez-Medina, Antonio Martinez-Riera

**Affiliations:** 1 Dpto. de Medicina Interna Hospital Universitario de Canarias La Laguna Tenerife, Canary Islands Spain; 2 Dpto. de Anatomia Patológica Hospital Universitario de Canarias La Laguna Tenerife, Canary Islands Spain

## Abstract

A middle-aged woman suffering from CMV pancreatitis and HIV positive was treated


*HPB Surgery*, 1992, Vol. 5, pp. 181–184
Reprints available directly from the publisher
Photocopying permitted by license only

© 1992 Harwood Academic Publishers GmbH
Printed in the United Kingdom

### CYTOMEGALOVIRUS-ASSOCIATED PANCREATITIS IN ACQUIRED IMMUNODEFICIENCY SYNDROME

EMILIO GONZÁLEZ-REIMERS[1], FRANCISCO SANTOLARIA-FERNÁDEZ[1], JUAN L. GÓMEZ-SIRVENT[1], RAFAEL MÉNDEZ-MEDINA[2] and ANTONIO MARTINEZ-RIERA[1]

*[1]Dpto. de Medicina Interna; [2]Dpto. de Anatomia Patológica. Hospital Universitario de Canarias, La Laguna, Tenerife, Canary Islands (Spain)*

*(Received 5 September 1991)*

**A middle-aged woman suffering from CMV pancreatitis and HIV positive was treated.**

KEY WORDS: Aids, pancreatitis, cytomegalovirus

Cytomegalovirus (CMV)-associated acute pancreatitis is a rare condition[1,2], although CMV inclusions in the pancreas may be seen in autopsy series of patients with AIDS[3,4]. CMV pancreatitis in these patients may present in an atypical fashion, and diagnosis may be obscured by accompanying diseases. We describe here a new case of this entity, in which pancreatic damage persisted for four months and the clinical picture, although compatible with acute pancreatitis at the beginning, was masked by superimposed disease during the clinical course.

A fifty-year-old was admitted to the Hospital with a four-day history of disorientation, mental obtundation and continuous vomiting. She had never consumed any kind of drugs, including ethanol, and her only sexual partner also denied other sexual contacts. She had been operated on (hysterectomy) three years before in another hospital (before Spanish law made obligatory HIV-antibody determination in blood donors). At that time she received a blood transfusion.

Physical examination on admission revealed a pale, undernourished and confused middle-aged woman, with profuse sweating and a blood pressure of 80/50 mm Hg. Examination of the oral cavity showed candidiasis. Chest radiography, lumbar puncture, cerebral computed tomography (CT), and blood and urine cultures at admission were negative, and routine urine and blood analysis were normal except for the following: serum amylase 1288 U/l, serum albumin 30 g/l, serum AST 89 U/l, ALT 98 U/l, serum glucose 411 mg/dl (22.8 mmol/l), LDH 558 U/l, platelet count, 87 × $10^9$/l (87000/$mm^3$). Abdominal ultrasound examination revealed a normal but hypo-echogenic pancreas, normal bile ducts, and absence of gallstones.

Treatment with amphotericin, parenteral nutrition, cephotaxime and netilmicin led to an improvement in her general condition; the patient began to eat, the serum amylase on the fourth day had returned to normal (187 U/l). On the 10th day she complained of fever and diarrhoea. Anaemia (haemoglobin 81 g/l) and leucopenia

Address correspondence to: Emilio Gonzalez-Reimers, Dpto. de Medicina Interna, Hospital Universitario de Canarias, La Laguna, Tenerife, Canary Islands

(WBC 2300 × $10^9$/l) developed, and serum amylase was raised (303 U/l). An abdominal CT scan was performed, showing multiple abnormal areas in the head of pancreas, which was slightly enlarged. At that time, liver function tests showed an alkaline phosphatase of 1188 U/l, gammaglutamyltranspeptidase 683 U/l, AST 103 U/l and ALT 143 U/l. This new episode improved after treatment, serum amylase returning to normal (188 U/l). A herpes simplex virus infection appeared on the upper lip, and she complained of progressive dysphagia and extense oropharyngeal candidiasis. This clinical picture was accompanied by fever, occasional diarrhoea and vomiting. An HIV antibody test was positive. The patient's general condition slowly worsened, with fever, progressive thrombopenia, leucopenia, and gastrointestinal bleeding. She died 4 months after admission. Serum amylase shortly before death was normal (88 U/l). Serum calcium and triglycerides were always within the normal range.

Postmortem examination of the pancreas showed patchy areas of necrosis, fibrosis and a chronic inflammatory infiltrate. Numerous characteristic cytomegalovirus inclusions were observed in the pancreas (Figure 1), lung and small intestine, but not in the liver, gallbladder or bile ducts.

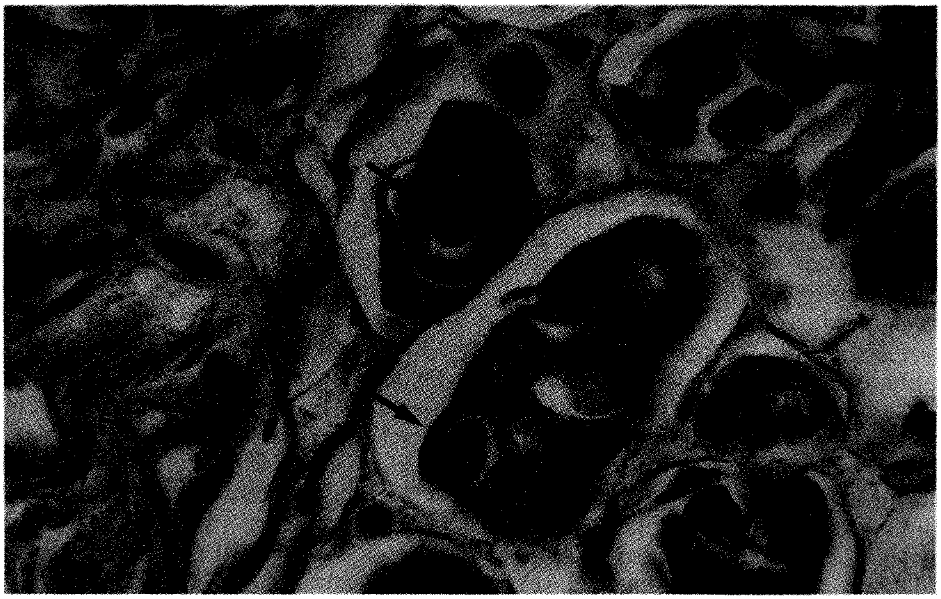

**Figure 1** Characteristic CMV inclusions in pancreatic acinar cells (arrows) (HE, 1000×).

Well-documented CMV pancreatitis has been rarely reported in immunosuppressed non-AIDS[5,6] or AIDS patients[1,2]. Ischaemia due to involvement of endothelial cells by CMV is proposed as the underlying pathogenic mechanism for the pancreatitis[1]. Diagnosis in most cases has been established only postmortem. In our case, although pancreatitis was promptly diagnosed, the clinical course of the disease became masked by superimposed complications resulting from immunosuppression. However, fat necrosis was still present 4 months after the initial attack.

No other aetiological factor was present: our patient had not been on drugs, she never drank alcohol, no gallstones were observed and there was no hypercalcemia or hyperlipidemia.

Thus, we conclude that this is a new case of CMV pancreatitis in a patient affected by AIDS. We agree with Wilcox and colleagues[1] that the antemortem diagnosis of this disease is difficult. In the present case, the absence of risk factors for HIV infection allowed us to think of acute pancreatitis at the time of admission. Had she belonged to a high-risk group, the diagnosis of pancreatitis would probably have been delayed or never made, as in the cases reported by Wilcox.

*References*

1. Wilcox, C.M., Forsmark, C.E., Grendell, J.H., Darragh, T.M. and Cedllo, J.P. (1990) Cytomegalovirus-associated acute pancreatic disease in patients with acquired immunodeficiency Syndrome. *Gastroenterology*, **99**, 263–267
2. Teixidor, H.S., Honig, C.L., Norsoph, E., Albert, S., Mouradian, J.A. and Whalen, J.P. (1987) Cytomegalovirus infection of the alimentary canal:radiologic findings with pathologic correlation. *Radiology*, **163**, 317–323
3. Bricaire, C., Marche, C., Zoubi, D., Saimot, A.G. and Regnier, B. (1988) HIV and the pancreas. *Lancet*, **i**, 65–66
4. Zazzo, J.F., Pichon, F. and Regnier, B. (1987) HIV and the pancreas. *Lancet*, **ii**, 1212–1213
5. Parham, D.M. (1981) Post-transplantation pancreatitis associated with cytomegalovirus (report of a case). *Human Pathol.*, **12**, 633–665
6. Iwasaki, T., Tashiro, A., Satodate, R., Sata, T. and Kurata, T. (1987) Acute pancreatitis with cytomegalovirus infection. *Acta Pathol. Jpn.*, **37**, 1661–1668

*(Accepted by S. Bengmark 5 September 1991)*

### INVITED COMMENTARY

Detailed post-mortem studies have shown disorders of the pancreas in half the patients dying of AIDS[1–3] but many of these were unsuspected during life. Acute pancreatitis can result from opportunistic infection of the pancreas, from drugs such as pentamidine or cotrimoxazole used to treat respiratory infection, or possibly from direct involvement of the pancreas by the human immunodeficiency virus (HIV) itself[4,5]. Chronic pancreatitis, diabetes and pancreatic neoplasia (lymphoma, Kaposi's sarcoma) may also give rise to symptoms in patients with HIV.

Cytomegalovirus is the commonest organism to affect the pancreas in AIDS patients, but the pancreatic involvement is often "silent" and occurs as part of a generalised CMV infection of the gut. Other opportunistic organisms include Cryptococcus sp., Toxoplasma gondii, Mycobacterium tuberculosis and Candida albicans. The case reported by Dr. Gonzalez-Reimers and his colleagues is unusual but not unique: there have been at least 3 other cases of CMV acute pancreatitis in AIDS patients[6,7]: When serum levels of amylase and lipase were measured twice-weekly in a group of 35 AIDS patients admitted to an intensive care unit, acute pancreatitis was diagnosed in no less than 16 (46%) and CMV was found at autopsy in 2 of the 8 who died[8]. However, macroamylasaemia may explain some cases of "biochemical" pancreatitis in patients with a disordered immune system[9].

Doctors looking after AIDS patients should also be aware of the existence of "AIDS cholangiopathy" and of papillary stenosis.

Several serological tests are available for the rapid detection of CMV antibodies, but a positive test does not necessarily mean active infection. Identification of CMV infection requires demonstration of a cytopathic effect in human diploid fibroblasts cultured with an appropriate fluid, such as blood, urine, saliva or perhaps pancreatic juice obtained at ERCP. If CMV infection were established or strongly suspected, there would be a case for adding ganciclovir to the usual supportive therapy for acute pancreatitis in an attempt to prevent a fatal outcome.

N. Bliouras
R.C.N. Williamson

*References*

1. Brivet, F., Coffin, B., Bedossa, P., Naveau, S., Petitpretz, P., Delfraissy, J.F. and Dormont, J. (1987) Pancreatic lesions in AIDS. *Lancet*, **i**, 570–571 (letter)
2. Bricaire, C., Marche, C., Zoubi, D., Saimot, A.G. and Regnier, B. (1988) HIV and the pancreas. *Lancet*, **i**, 65–66 (letter)
3. Dowells, S.F., Moore, G.W. and Hutchins, G.M. (1990) The spectrum of pancreatic pathology in patients with AIDS *Mod. Pathol.*, **3**, 49–53
4. Torre, D., Montomari, M., Fiori, G.P., Dietz, A. and Sampietro, C. (1987) HIV and the pancreas. *Lancet*, **ii**, 1212 (letter)
5. Clas, D., Falutz, J. and Rosenberg, L. (1989) Acute pancreatitis associated with HIV infection. *Can. Med. Assoc. J.*, **140**, 823
6. Joe, L., Ansher, A.E. and Gorbin, F.M. (1989) Severe pancreatitis in an AIDS patient in association with cytomegalovirus infection. *South. Med. J.*, **82**, 1444–1445
7. Wolf, P., Reiser, J.R., Fellow, J.Ee. and Haghighi, P. (1989) Pancreatitis in patients with AIDS presumptively due to CMV. *J. Clin. Lab. Anal.*, **3**, 152–155
8. Zazzo, J.F., Pichon, F. and Regnier, B. (1987) HIV and the pancreas. *Lancet*, **ii**, 1212–1213 (letter)
9. Schwartz, M.S. and Brandt, L.J. (1989) The spectrum of pancreatic disorders in patients with the acquired immune deficiency syndrome. *Am. J. Gastroenterol.*, **84**, 459–462

